# Arthralgia and blood culture-negative endocarditis in middle Age Men suggest tropheryma whipplei infection: report of two cases and review of the literature

**DOI:** 10.1186/s12879-015-1078-6

**Published:** 2015-08-18

**Authors:** Anthony Alozie, Annette Zimpfer, Kerstin Köller, Bernd Westphal, Annette Obliers, Andreas Erbersdobler, Gustav Steinhoff, Andreas Podbielski

**Affiliations:** Department of Cardiac Surgery, University Hospital Rostock, Schillingallee 35, 18057 Rostock, Germany; Institute of Pathology, University Hospital Rostock, Strempelstr. 14, 18055 Rostock, Germany; Institute of Medical Microbiology, Virology and Hygiene, University Hospital Rostock, Schillingallee 70, 18055 Rostock, Germany

**Keywords:** Infective endocarditis, Biological therapy, Tropheryma whipplei, Rheumatoid arthritis, Arthralgia

## Abstract

**Background:**

Whipple’s disease is a rare, often multisystemic chronic infectious disease caused by the rod-shaped bacterium *Tropheryma whipplei*. Very rarely the heart is involved in the process of the disease, leading to culture-negative infective endocarditis. Up to 20 % of all infective endocarditis are blood culture-negative and therefore a diagnostic challenge. We present two unusual cases of culture-negative infective endocarditis encountered in two different patients with prior history of arthralgia. A history of rheumatic arthritis or even a transient arthralgia should put *Tropheryma whipplei* on the top of differentials in patients of this age group presenting with culture-negative infective endocarditis, especially in cases of therapy resistance to antirheumatic agents.

**Case presentation:**

The first patient was a 55 year-old Caucasian male with culture-negative Whipple-related adhesive pericarditis and endocarditis of the aortic valve. Importantly, the patient reported a 15-year history of therapy resistant sero-negative migratory polyarthritis. Aortic valve endocarditis developed during treatment with tocilizumab. The second patient was a 65-year-old male patient with no prior history of the classic Whipple’s disease who presented with a culture-negative aortic valve endocarditis. His past medical history revealed episodes of transient arthralgia, which he was not treated for however, due to the self-limiting nature of the symptoms. Both patients underwent aortic valve replacement surgery. During surgery, pericardectomy was necessary in the first patient due to adhesive pericarditis. Post surgery both patients were started on long-term treatment with trimetoprim-sulfamethoxazol. At 1-year follow-up of both patients, echocardiographic and clinical assessment revealed no signs of persistent infection. Both men reported negative history of arthralgia during the one year period post surgery.

**Conclusion:**

*Tropheryma whipplei* culture negative-infective endocarditis is an emerging clinical entity, predominantly found in middle-aged and older men with a history of arthralgia. These data highlight the need for ruling out Whipple’s disease in patients with a history of arthralgia prior to initiation of biological agents in treatment of rheumatoid arthritis. There is also a need to assess for *Tropheryma whipplei* in all patients with culture- negative infective endocarditis.

## Background

Whipple’s disease is a rare, often multisystemic chronic infectious disease caused by the rod-shaped bacterium *Tropheryma whipplei* [1]. A symptomatic disease only develops in patients with partially defined immunologic defects, i.e., Th1/Th2-imbalances [2]. The disease classically manifests with diarrhea, weight-loss and arthropathy. Cardiac involvement is rarely part of the primary syndrome [3]. Blood cultures may be negative in Whipple’s endocarditis [4]. Other manifestations of Whipple’s disease are frequently absent in patients with Whipple’s endocarditis [4]. However, with the availability of molecular diagnostic tools such as polymerase chain reaction (PCR), more cases are increasingly recognized [5, 6]. The increased recognition rate led to the insight that Whipple’s endocarditis can occur without other classical manifestations of Whipple’s disease [4]. In Germany, the reported incidence rate for *T. whipplei* endocarditis is about 6 % of all infectious cases: *T. whipplei* was the fourth most frequent pathogen found among 255 cases of endocarditis with an etiologic diagnosis and was the most common pathogen associated with blood culture–negative endocarditis [19].

## Case presentation

### Case 1

A 55-year old Caucasian male presented with progressive muscle weakness, unintended weight-loss over a period of 6 months, malaise and exercise dependent dyspnea over a period of 4 weeks. He denied fever or chills but reported episodic light-headedness and shortness of breath. Holter ECG revealed intermittent second and third degree atrio ventricular block. Significant in his past medical history was a 15-year intermittent joint swelling and early morning stiffness, affecting predominantly both ankle joints and the wrists, which led to the diagnosis of sero-negative rheumatoid arthritis. Therapeutic trials with different antirheumatic agents including Methotrexate, Adalimumab, Cyclosporin A, Etanercept and lastly Tocilizumab did not lead to any sustainable remission. Prior to diagnosis of aortic valve endocarditis he was receiving 10 mg prednisolon orally daily, with moderate success. A positive quantiferon-TB test was reported 1 year prior to current admission. At that time, despite lacking clinical signs of active tuberculosis, Isoniazid and Rifampicin therapy was initiated, but stopped shortly thereafter due to severe adverse effects on the patient. Until the current admission, he remained without obvious clinical signs of active tuberculosis.

**Fig. 1:**
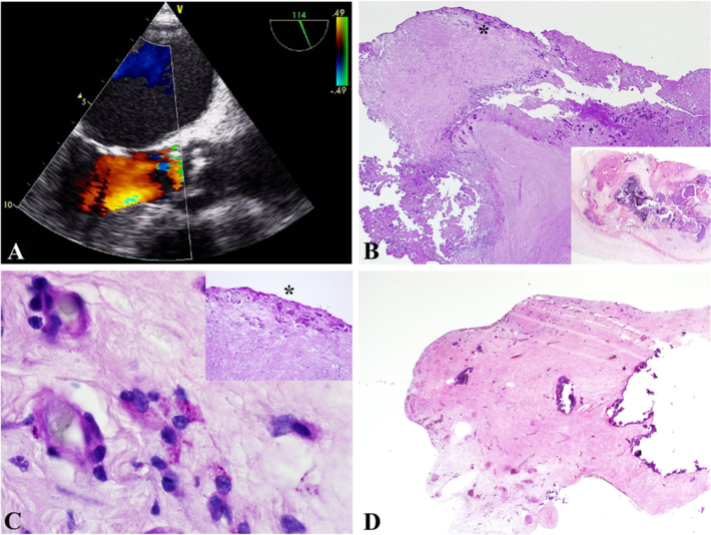
**a**–**d**: Whipple endocarditis and pericarditis in a 55-year old patient. **a**. TEE showing a severe degenerative aortic valve and aortic valve vegetations with aortic regurgitation III°. **b**. Severely degenerative and calcific aortic disease with chronic ulcerations (Pas, 2x, inset: hematoxillin&eosin, 2x). *labels the area visualized in 1C. **c**. Demonstration of Pas-reactive foamy macrophages, which contained T. whipplei particles. Note: the macrophages are seen in relation to capillaries (Pas, 100x, inset: Pas, 10x). **d**. Pericardium with extensive sclerosis and calcification (hematoxillin&eosin, 2x)

Complete laboratory panels on admission revealed C-reactive protein 90 mg/l, leucocytes count 13 × 10 ^9^/l and procalcitonin 0.11 g/l. No anemia or elevated markers of myocardial ischemia and liver pathology were apparent. On physical examination, a 3/6 early diastolic decrescendo murmur best heard at the left intercostal space was appreciated. A chest radiograph showed mild cardiomegaly and pulmonary edema. Transthoracic and transesophageal echocardiograms demonstrated 13 × 10 mm mobile aortic valve vegetations on the ventricular surface of the aortic valve and severe aortic regurgitation (Fig. 1a). Because of six negative blood cultures, a presumptive diagnosis of culture-negative aortic and mitral valve endocarditis was made and empiric antibiotic therapy with ampicillin/sulbactam and gentamycin was started. Expedited indication for aortic valve replacement surgery was made and the patient was transferred to the heart surgery unit for operation, which was carried out 2 days later.

He underwent tissue aortic valve replacement and intrasurgical transaortic direct visualization of the mitral valve. The mitral valve was merely mildly degenerative but competent and without evidence of endocarditis. After sternotomy, the pericardial tissue was found to be predominantly adherent to the epicard, requiring extensive adhesiolysis before installation of the heart lung machine. The aortic valve was severely degenerated and calcified. At first, no macroscopic evidence of endocarditis was noticed. However, free floating vegetation was visualized on the subvalvular surface of the aortic valve attached between the left and right commissural junction. Specimens from both the aortic valve and pericardium were taken for microbiological and histopathological analysis. Otherwise, surgery progressed without complications and the patient was transferred to the intensive care unit for overnight monitoring.

The antibiotic treatment regimen was complemented with vancomycin and rifampicin prior to surgery and was continued until the fourth post surgical day. Polymerase chain reaction (PCR) results revealed T*ropheryma whipplei* as the causative agent. This was confirmed by histopathological visualization of PAS and CD-68 positive macrophages on both aortic and pericardial tissues (Fig. 1b–d). A diagnosis-adapted scheme of the antibiotic regimen was immediately installed, comprising ceftriaxon (4 weeks), meropenem (14 days), and Trimethoprim/Sulfamethoxazol, which was to be continued for 1–2 years depending on clinical and paraclinical response to this therapy regimen. In-hospital stay was prolonged due to persistent grade III° atrial ventricular block necessitating transvenous implantation of a DDD-pacemaker on the 7^th^ post-surgical day. Then, the patient was transferred to a neighboring primary care center for further intravenous antibiotic treatment and monitoring before starting three weeks of rehabilitation.

At one-year follow-up, prednisolon dosage has been tapered and therapy terminated, without current clinical evidence of active arthritis. Regular echocardiographic and clinical evaluation remained stable and positive except for a persistent CRP values ranging between 10 and 25 mg/l but normal leucocytes. Gastroenteroscopic biopsy analysis revealed no evidence of persistent Whipple’s disease. Continued regular echocardiographic and clinical check-ups remain imperative for this patient.

### Histopathology

Histologic examination of the resected aortic valve showed a severely degenerative calcific valvular disease with a chronic resorptive inflammation and superficial chronic erosions and ulcerations (Fig. 1b). With PAS reagent, foamy macrophages with granular intracellular PAS positive material, characteristic of *T. whipplei* infection, were demonstrated (Fig. 1c). Separately submitted pericardial tissue showed a severely sclerosed pericardium with multiple calcifications (Fig. 1d). There was only a mild chronic inflammation present, but foamy macrophages with typical PAS-positive inclusions indicating the presence of T. whipplei could also be demonstrated.

### Microbiology

Processing of blood cultures followed the established standards of the German Society for Hygiene and Microbiology (DGHM). The microbiology laboratory of the Rostock University Medicine is accredited according to DIN EN ISO 15183 for these tests as well as for the subsequently described PCR examinations. DNA extraction was performed with the Qiagen DNA Mini Kit (QIAGEN, Hilden, Germany) according to the manufacturer’s protocol. Nucleic acid concentration was measured using a biophotometer (Eppendorf, Hamburg, Germany). For 16S rDNA PCR primers 16S8_27 and 16S_907 and polymerase moltaq (molzym) was utilized [7, 8]. The following reaction conditions were chosen: (i) 15 min at 94 °C; (ii) 30x [1 min at 94 °C, 1 min at 50 °C, 1 min at 72 °C]; (iii) 5 min at 72 °C [7, 8]. PCR products were determined by gel electrophoresis, purified with the NAT CLEAN-UP/NUCLEOSPIN® EXTRACT II (Machery-Nagel, Düren, Germany) and subsequently sent to Microsynth Sequencing Device (Göttingen, Germany) for the actual sequencing reaction. Sequence analysis was performed using NCBI nucleotide blast search (http://www.ncbi.nlm.nih.gov), resulting in identification of *T. whipplei.*

### Case 2

**Fig. 2:**
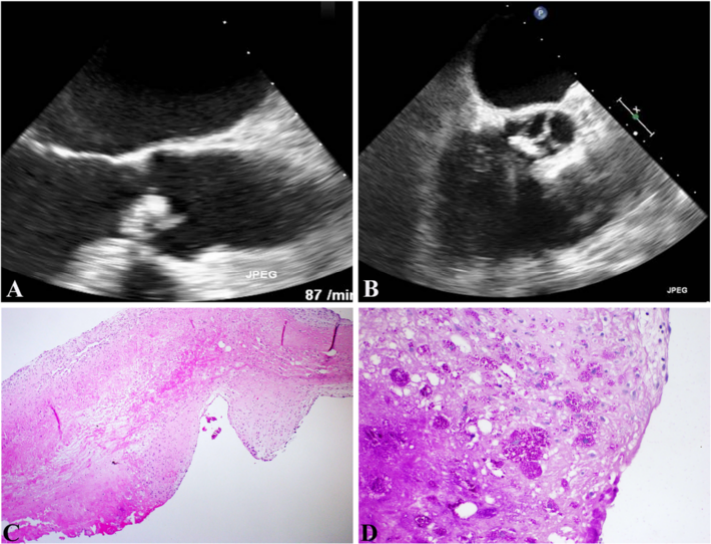
**a**–**d**: Whipple endocarditis in a 65- year- old patient. **a**/**b**. TEE displaying degenerative aortic valve with vegetations. **b**. Mild degenerative aortic valve changes with pronounced edema, fibrin insudation and superficial collections of foamy macrophages (haematoxillin&eosin, 4x). **d**. Demonstration of Pas-reactive foamy macrophages, which contained T. whipplei particles (Pas, 20x)

A 65-year- old male patient presented to his family physician for routine check-up with feelings of exhaustion over the previous 2- months, mild exercise induced dyspnea and occasional palpitations. On cardiac auscultation a 2/6 early diastolic decrescendo murmur best heard at the third left intercostals space radiating along the left sternal border was appreciated. He reported yearly travel to Denmark and Sweden for the purpose of holidays, but denied any close contact to livestock, pets or poultry. Laboratory test on admission revealed 9 × 10 [9]/L white blood cells, 31 mg/l c-reactive protein and 0.074 ng/ml procalcitonin respectively. All other laboratory values including haemoglobin, liver and renal parameters were within normal range. A chest x-ray film revealed bilateral accentuated perihilar haze and mild cardiomegaly. Abdominal sonography only revealed a mild splenomegaly. Transthoracic echocardiographic exploration revealed 1 cm × 1 cm vegetation over aortic surface of the aortic valve (Fig. 2a–b). Aortic regurgitation grade III° with reduced left ventricular ejection fraction (40 %) was also appreciated. He was started on a triple antibiotic combination of Rifampicin (2 × 600 mg i.v., Gentamicin i.v. according to blood levels and Sulbactam/Ampicilin 2 × 3 g i.v.). Two sets of blood cultures remained negative. During surgery for aortic valve replacement, all valve leaflets with exception of the valve ring were involved in the endocarditic process, with severe destruction of all valves. Indication for a 21 mm trifecta tissue valve (St Paul, Minn) was made after excision of the diseased valve. Post surgery the patient was transferred to the ICU and after an uncomplicated ICU stay he was transferred to the normal ward. Further hospitalisation was uncomplicated and the patient was discharged to our cardiology unit on the 7th day post surgery. Polymerase chain reaction (PCR) results 10 days post surgery revealed T*ropheryma whipplei* as the causative agent. This was confirmed by histopathological visualization of Periodic Acid Schiff (PAS) and CD-68 positive macrophages on the aortic tissues (Fig. 2c–d). A diagnosis-adapted scheme of the antibiotic regimen was immediately installed comprising ceftriaxon (4 weeks), meropenem (14 days), and Trimethoprim/Sulfamethoxazol, which was to be continued for 2 years depending on clinical and paraclinical response to this therapy regimen. At 6-month- follow up, echocardiographic and clinical evaluation remained stable with normal infective parameters. During the six-month follow- up post surgery, the patient revealed that prior to his hospitalization for endocarditis treatment he had experienced 4 episodes of self-limiting polyarthralgia involving his toes, ankles and fingers of over a period of two years. Though he sought a medical attention, it was neither treated nor followed up extensively due to its self-limiting nature, lasting only a couple of days with mild swelling of the involved joints. He reported complete remission since surgery and ongoing treatment of infective endocarditis. Gastroenteroscopic biopsy analysis was pre and post operatively not necessary due to absent gastro intestinal symptoms in our patient, in particular: no weight loss or diarrhea was reported. Continued regular echocardiographic and clinical check-ups remain imperative for this patient.

### Histopathology

Histologic examination of the submitted specimen showed severely degenerative calcific valvular disease with a chronic resorptive inflammation (Fig. 2c–d). With PAS reagent, foamy macrophages with granular intracellular PAS positive material, characteristic of *T. whipplei* infection, were demonstrated (Fig. 2c–d).

### Microbiology

DNA analysis performed as stated above revealed *T. whipplei* as causative agent.

### Discussion and literature review

Whipple’s disease is a rare chronic, often multisystemic disease, which was first described by George Hoyt Whipple in 1907 [9]. But, it was only after 1992 that the causative agent, a rod-shaped, gram-positive bacterium, *Tropheryma whipplei,* was identified by sequencing its ribosomal RNA (rRNA) gene [10]. In spite of its bacterial etiology, Whipple’s disease is a non-readily-contagious condition, which mainly affects patients with a Th1/Th2 imbalance [2]. Chronic asymptomatic carriage of *T. whipplei* has been found in stool specimen of human, especially those exposed to sewage water [11]. However, the infectious potential of *Tropheryma whipplei* is a moot point [12]. There is evidence that person-to-person close contacts either by faeco-oral or by oral to oral routes support bacterial transmission [13]. General hygiene practices to prevent faeco-oral transmission are therefore highly recommended for vulnerable population groups, since predisposing immunologic or genetic factors would favour development of Whipple’s disease in the long run [14, 15]. After contamination, patients may develop acute infection with specific antibody response. Thereafter, two principal ways of dealing with the infectious agent are envisioned: patients displaying a strong immune response and harbouring specific antibodies for at least 5 years eventually eliminate *T. whipplei* from their intestines, while patients developing no or only a minor antibody response will suffer from subacute or chronic infections, including classic Whipple’s disease [16].

Whipple’s disease has a variable symptomology, explainable by *T. whipplei* invasion into various tissues and subsequent incorporation by tissue macrophages, which are unable to eliminate the bacteria [4]. In Whipple’s endocarditis, the infection is slowly progressive, similar to that seen in Q fever and bartonellosis-induced culture-negative endocarditis, and a prominent fibrosis with mild chronic inflammation is seen histopathologically [4]. In most cases, the cardinal features, i.e., diarrhea, weight loss and arthralgia are present. However, in the absence of this symptom-complex, diagnosis may be extremely difficult, elusive and delayed due to the absence of an easily available and reliable serological or molecular method to detect this microorganism in blood [17, 18].

Infective endocarditis during classical Whipple’s trio (arthropathy, weight-loss and diarrhea) due to *T. whipplei* is rare and its diagnosis could be difficult if it presents without any prior history suggesting Whipple’s disease, in which case blood cultures remain negative [18, 19]. Most information on this entity has accrued from occasional case reports and case series [18]. In fact, the prevalence of infective endocarditis due to *T. whippelei* could be higher in patients treated for culture-negative endocarditis [19]. In contrast to previous reports, Geiβdörfer et al. found *T. whipplei* to be the fourth most frequent cause of bacterial endocarditis (6.3 %) and the most frequent cause of culture-negative endocarditis [18, 19]. Out of 16 patients with *Tropheryma whipplei* endocarditis, 14 lacked gastrointestinal symptoms prior to surgical treatment [19]. According to Fenollar et al., the following characteristics may aid in the recognition of possible Whipple associated infective endocarditis in the absence of classical diagnostic criteria: T. whipplei endocarditis occurs mainly in Caucasian men, who are about 50 years of age with cardial symptoms such as heart failure, acute ischemic stroke and peripheral arterial embolism [5]. These patients might have complained about arthralgia for several years and might have recently received immunosuppressants [5]. Arthralgia might be subtle and is only noticed after a careful clinical investigation [5]. They strongly suggested inquiring for the presence of arthralgia since the combination of infective endocarditis and arthralgia suggests *T. whipplei* infection [5]. This is in line with the history of our first patient, as we observed a possible association between exposure to certain immune-modulatory agents and exacerbation of otherwise under diagnosed Whipple’s disease. Our patient had sero-negative rheumatoid arthritis for approximately 15 years and only complained of unintended weight loss over a period of 6 months prior to diagnosis of infective endocarditis. No other symptoms were recorded, until infective endocarditis struck. The second patient reported none of the symptoms that would suggest *T. whipplei* involvement in his ailment. However, during follow up at 1-year post surgery, a comprehensive interview revealed that the patient had a benign, self limiting arthralgia, which was not medically attended to, due to the subtle nature of his symptoms.

So far, little attention has been given to consequences of *T. whipplei* infection exacerbation during therapy with biological agents, a potentially fatal coincidence. Table 1 presents reported cases and case series of patients with symptoms initially misinterpreted until clinical deterioration under various immune-modulatory agents, especially TNFα antagonists, facilitating a more fulminant course of *T. whipplei* infection [20–33].

**Table 1:** Reports indicating alteration of the course of subacute Whipple’s disease during therapy with biological agents

Nr.	Reports Indicating Potential Fatal Complications Of Biological Therapy During Sero-negative Rheumatoid Arthritis.							
	Author	Journal	Yr of Pub	Nr. of Pat.	Initial symptoms/Duration	Therapy	Results	Conclusion/Finding
[20]	Mahnel R. et al.	Am. J. Gastroenterol.	2005	27				Immunosuppressiva lead to earlier onset of diarrhea
[21]	Kneitz C. et al.	Scand. J. Rheumatol.	2005	1		Infliximab/MTX	Rapid weight loss Erythema nodosum diarrhea LN enlargement sigmoido-vesical fistula	Infliximab seems to increase the risk of exacerbation of WD
[22]	Razonable R.R. et al.	Transpl. Infect. Dis.	2008	1	Kidney Transplant and migratory poly arthritis, weight loss, GIT-symptoms for years	Azathioprim/Prednison	Chorioretinitis and Vitreitis	Tropheryma whipplei DNA in vitrous fluid and peripheral blood
[23]	Kremer AE. et al.	Z. Gastroenterolog.	2008	1	SNRA/4 yrs	Adalimumab/Leflunomid	Septic fever severe arthralgia weight loss	Immunmodulatory therapies, TNF blockers and Corticosteroids may lead to exercerbation of subacute, undiagnosed Whipples Disease
[24]	Spoerl D. et al.	Orphanet. J. Rare Dis.	2009	1	Multisegment spondylitis	TNF-a	Worsening back pain	TNF-a treatment worsened spondylodiscitis, leading to diagnosis of T. Whipplei from rebiopsy of vertebral specimen
[25]	Lagier J-C et al.	Medicine	2010	113	5 of 16 patients with endocarditis as initial symptoms had immunosuppressive treatment	Corticosteroids 50 (43 %) TNF-a antagonists 16 (14 %) Others 16 (14 %) Previous immuno suppressive Treatment 56 (50 %)	32 patients (28 %) experienced aggravation of various symptoms after immunosuppressive therapy	Patients with inflammatory rheumatoid disease who experience severe general involvment should be screened for T. Whippelii or the therapy is inneffective against polyarthritis
[26]	Hoppe E. et al.	Joint bone Spine	2010	5	RA(2), AS(2), SA(1)	TNF a antagonists	Failure to control the disease and other symptoms	Biological therapy probably worsened pre existing whipples disease
[27]	Vancsa A et al.	Joint Bone Spine	2010	1	Seronegative Oligoarthritis	Eternecept	Endocarditis	DMARDs resistant arthritis should prompt thourough search T.whippelii prior to initiation of TNF-a antagonists
[28]	Hmamouci I et al.	J. Rheumatol.	2010	1	Ankylosing spondylitis	Eternecept	Scurvy	Eternecept probably modified the cytokine environment and thus favoured exercerbation of whipples disease
[29]	Daien C.I. et al.	Rheumatology	2010	1	B-27- negative Ankylosing spondylitis	Eternecept	Endocarditis	Report of the first case of t. Whippeliis endocarditis, potentially induced by TNF-a antagonist therapy
[30]	Gaddy J.R.	Rheumatology	2012	1	Back pain; Arthritis	Various TNF a Inhibitors	Fever, migratory arthritis	Clinical deterioration despite TNF a antagonists lead to thorough search and T. Whipelli was found
[31]	Sparsa L et al.	La Revue de medecine interne	2013	2	Spondyloarthritis (both patients)	Eternecept/adalimumab (Both patients)	peristent elevated acute phase reactants	Whipples disease should be suspected in patients with treated with TNF a antagonists who do not improve during inflammatory rheumatism
[32]	Marth T.	World J. Cardiol.	2014	41vs 61vs1059	Arthritis	TNF-a/41 patients	12.2 % vs 1.6 % vs 0.16 % endocarditis rates	TNF-a triggered severe whipples disease complications particularly endocarditis
[33]	Marth T.	Aliment. Pharmacol. Ther.	2015	41	Arthritis, weight-loss, Diarrhea	Various TNF-a Inhibitors	Fever, septic temperatures (n, 16), Tropheryma whipplei septicemia (n, 6), Endocarditis (n, 5) etc.	In case of doubt, Whipples disease should be excluded before therapy with TNF-a

## Conclusion

We strongly advocate intensive search for a potential occult Whipple’s disease in patients with therapy resistant sero-negative rheumatoid arthritis prior to introduction of immune modulatory therapies. *T. whipplei* should always be ruled out in patients who present with unexplained transient arthralgia irrespective of duration, frequency and severity. Exacerbation of subacute Whipple’s disease should be added to the list of possible side effects of therapy with these agents, especially TNFα antagonists.

### Consent

Written informed consent was obtained from the patients for publication of the Case report and any accompanying images. A copy of the written consent is available for review by the Editor of this journal.

## Abbrevations

AS: Ankylosing spondylitis
CNS: Central nervous system
DMARD: Disease-modifying anti rheumatic drugs
GIT: Gastrointestinal tract
LN: Lymph node
NSAID: Non-steroidal anti-inflammatory drug
pub: publication
RA: Rheumatoid arthritis
SA: Spondylarthritis
SNRA: Seronegative rheumatoid arthritis
yr: year
